# Reconstruction of functional human epidermis equivalent containing 5%IPS-derived keratinocytes treated with mitochondrial stimulating plant extracts

**DOI:** 10.1038/s41598-022-13191-4

**Published:** 2022-05-31

**Authors:** Marielle Moreau, Christophe Capallere, Laurent Chavatte, Christelle Plaza, Céline Meyrignac, Karl Pays, Bruno Bavouzet, Jean-Marie Botto, Carine Nizard, Anne-Laure Bulteau

**Affiliations:** 1 LVMH Recherche. Life Science Department, 185 Avenue de Verdun, 45800 Saint Jean de Braye, France; 2 Advanced Skin Research & Bioengineering Department, Ashland, Global Skin Research Center, Sophia Antipolis, France; 3 Centre International de Recherche en Infectiologie, CIRI, 69007 Lyon, France; 4 Institut National de La Santé Et de La Recherche Médicale (INSERM) Unité U1111, 69007 Lyon, France; 5 Ecole Normale Supérieure de Lyon, 69007 Lyon, France; 6 Université Claude Bernard Lyon 1 (UCBL1), 69622 Lyon, France; 7 Unité Mixte de Recherche 5308 (UMR5308), Centre National de La Recherche Scientifique (CNRS), 69007 Lyon, France

**Keywords:** Reprogramming, Skin stem cells, Energy metabolism

## Abstract

Reconstructed human epidermis equivalents (RHE) have been developed as a clinical skin substitute and as the replacement for animal testing in both research and industry. KiPS, or keratinocytes derived from induced pluripotent stem cells (iPSCs) are frequently used to generate RHE. In this study, we focus on the mitochondrial performance of the KiPS derived from iPSCs obtained from two donors. We found that the KiPS derived from the older donor have more defective mitochondria. Treatment of these KiPS with a plant extract enriched in compounds known to protect mitochondria improved mitochondrial respiration and rendered them fully competent to derive high-quality RHE. Overall, our results suggest that improving mitochondrial function in KiPS is one of the key aspects to obtain a functional RHE and that our plant extracts can improve in this process.

## Introduction

The regeneration or replacement of an irreversibly damaged organ that recapitulates the full-spectrum of its inherent physiological properties is an enduring challenge in modern medicine. The discovery that somatic cells can be reprogrammed into pluripotent stem cells (iPSCs) is a revolution for the field of regenerative medicine and tissue engineering^1^. With adequate culturing conditions, the iPSCs, endowed with unlimited proliferative capacities and extensive differentiation potential, can be coaxed to develop into a wide range of cell types^2,3^, with properties that physiologically resemble those of the original organ or tissue^4^, such as the skin. Skin is the largest human organ. Amongst its many essential functions, skin serves as one of the most effective barriers against the entry of harmful microbes or chemicals, but it is also an immense vehicle to transport active and functional pharmaceutical agents into the body. In parallel to skin regeneration research, the recent advance in 3D reconstruction of human skin equivalent (RHE) brought forth innovative clinical and pharmaceutical applications: RHEs are not only developed as sophisticated clinical skin replacements for burn victims, they also serve as versatile and effective models to replace animal testing in the pharmaceutical and cometic industry (reviewed in^5^). Such ingenious tissue engineering feat also relies on the iPSCs^1^.

For example, to evade transplant rejection, the emerging strategy is to generate a tissue/organ from a proliferative cell source bearing the same immune identity as the recipient. Human induced pluripotent stem cells are an ideal choice in this regard. In recent years, several RHE models have been developed to mimic the natural skin. One of the most prevalent methods to obtain RHE is to direct the iPSCs to differentiate into epidermal keratinocytes (KiPS) to generate a stratified epidermis^2^. In this case, iPSCs can be derived from the patient’s keratinocytes or fibroblast, but fibroblast has superior proliferative potential. By comparison, within the isolated keratinocytes, only a very small pool of cells can proliferate. Another major advantage of using fibroblast as the iPSC source is their easier accessibility and simpler maintenance in culture. RHEs based on KiPS are used to model after several human skin pathologies, including different skin cancers (reviewed in^5^); by incorporating a patient’s own genetic signature, they hold great potential in personalized medicine to yield tailor-craft individual therapeutic combinations based on ex vivo drug or treatment screen.

However, before the KiPS can be used to generate RHE, they must pass strict quality control. Many investigations have focused on scrutinizing the proliferative, differentiation and reverse-aging aspects of these cell. The studies examining the mitochondrial performance of KiPS are sparse by comparison^6^. Mitochondria are cytoplasmic organelles that produce adenosine triphosphate (ATP) to fuel the cell’s energetic demands through the process of oxidative phosphorylation. Besides acting as the powerhouse of a cell, mitochondria also play essential roles in cellular processes such as steroid metabolism, calcium homeostasis and apoptosis^7^. Mitochondrial function is repressed in iPSCs^8^. Structural and functional analyses of the mitochondria within the iPSCs reveal an immature network and low abundance compared to terminally differentiated cells^9^. Indeed, reprogramming of somatic cells to a pluripotent state leads to changes in cells physiology marked by the induction of a metabolic shift from oxidative phosphorylation to glycolysis^10^, as rapidly dividing cells rely on a glycolysis-based metabolism in order to adapt to their niche, a microenvironment characterized as an either hypoxic or low-oxygen environment^11^. Therefore, the observed metabolic shift from oxidative phosphorylation to anabolic glycolysis in iPSCs is regarded one of the most important prerequisite to maintain pluripotency^8^. However, the factors that control this metabolic shift are yet to be fully characterized. During iPSCs differentiation into somatic cells, mitochondria become mature and fully active as their metabolism switches from anaerobic glycolysis to oxidative phosphorylation^10,12–14^. Therefore, understanding the distinct metabolic properties of human pluripotent stem cells in comparison to their differentiated counterparts can be of crucial importance. Moreover, mitochondrial metabolism has been shown to regulate of keratinocyte differentiation in vivo^6^. In this study, we isolated normal fibroblasts from volunteer human donors to derive iPSCs, differentiated them into keratinocytes to reconstruct a 3D human epidermal (RHE) model, and characterized the metabolic and mitochondrial features of these cells. We first demonstrate that the mitochondria in KiPS switch from glycolysis to an oxidative phenotype, and that treating the KiPS with a mixed plant extract enriched in compounds beneficial to mitochondria function improve mitochondria performance. Finally, we succeeded in generating a fully functional RHE of optimal quality with a minimum input of the plant extract-treated KiPS. Our results thus provide a novel model for metabolic studies in reconstructed 3D human skin.

## Results

### Mitochondrial activity is repressed in iPSCs and restored in KiPS

**Figure 1:**
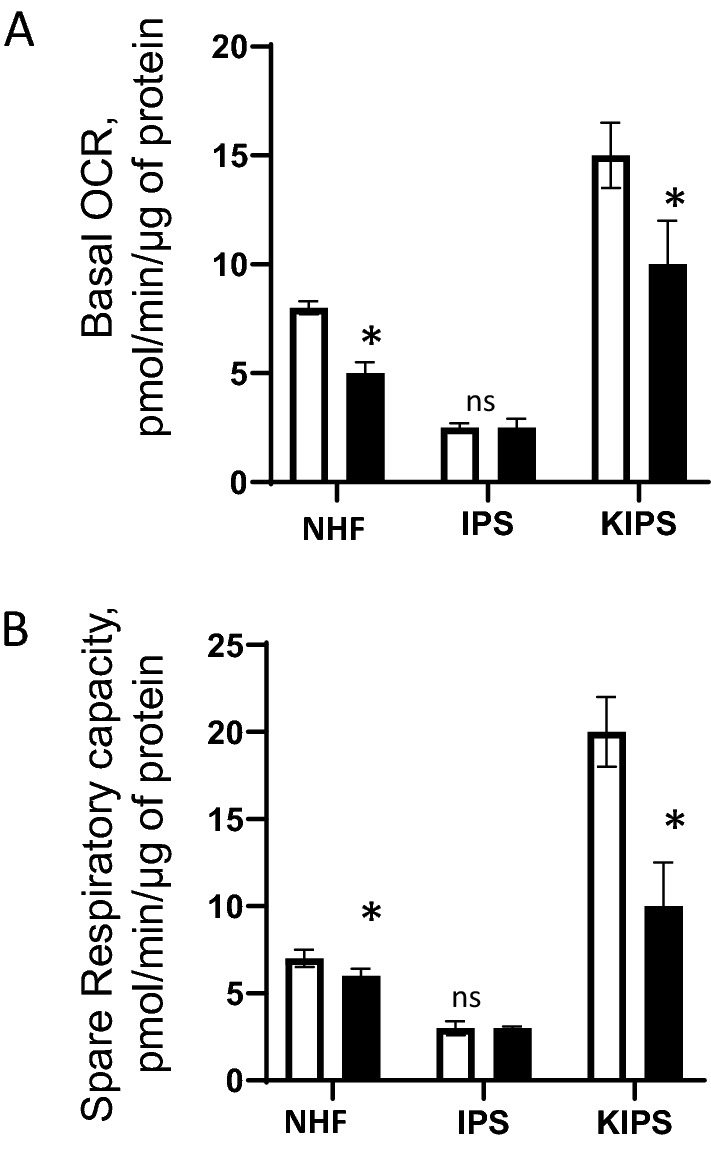
Mitochondrial contribution to the energetic metabolism of fibroblasts and their differentiated counterparts, iPSCs and KiPS. (**A**) The comparison of basal respiration results from Seahorse XF96 analyzer. Fibroblasts (NHF) from a 20-year old (white bars) and a 40-year old donor (black bars) were derived into iPSCs (IPS) and then these cells were differentiated into KiPS (KIPS) (see Materials and Methods). KIPS were analysed at day 8 after their differentiation (n = 2 experiments and 5 replicates). Mitochondrial inhibitors were sequentially injected; and basal respiration, which is the difference between the basal OCR and the oligomycin repressed OCR was determined. (**B**) Spare respiratory capacity is the calculated difference between the FCCP-induced OCR and the basal OCR. Statistical significance was determined by a Student’s *t* test. **P < 0.01 and *P < 0.05, errors bars: SEM.

To comprehensively compare the mitochondrial activity profiles in the iPSCs and their differentiated counterparts, we first isolated the normal human fibroblasts (NHF) from a 20-year old and a 40-year old donor, and transduced these NHFs with the non-integrative Sendai viral particles, which safely and effectively introduced four key genetic elements necessary to reprogram somatic cells into iPSCs. Upon the verification of the pluripotency, the iPSCs were induced to differentiate into two separate KiPS (KiPS-20 and KiP-40) lines by a first treatment with retinoic acid and BMP4, and a subsequent calcium exposure for terminal differentiation. Having successfully obtained the three different types of skin cultures, we set out to compare their mitochondrial bioenergetic profiles with the Seahorse technology that can concomitantly measure respiration (oxygen consumption rate [OCR]) and glycolysis (Fig. 1)^15^, by the incorporation of various mitochondrial inhibitors whose specific and distinct functions enable us to assess different bioenergetic parameters. Oligomycin is an ATP synthase inhibitor whose action allows us to derive Basal OCRs (measure of OXPHOS), which represent the amount of oxygen consumption linked to ATP synthesis in the mitochondria and is calculated as the difference between the average basal level of oxygen consumption before and after oligomycin treatment. Consistent with previous studies^16^, we found that in the NHFs from the younger donor, basal OCR was higher compared to that from the older donor (Fig. 1A). Reprogramming of these fibroblasts into iPSCs altered their mitochondrial function and induced a glycolytic shift, since in both the young and the aged iPSCs, the basal OCRs were highly reduced compared to their parental somatic NHFs. Notably, the rate of respiration of the older donor-derived iPSCs was similar to that of the young donor-derived iPSCs. Differentiation of iPSCs into keratinocytes resulted in a large and significant increase in the OCR, indicating that the differentiating keratinocytes switched to an oxidative phenotype, as increased oxygen consumption rate reflects the high energy demand required for lineage commitment, differentiation and maturation^17^. FCCP is a mitochondrial inhibitor that dissipates the proton gradient, and therefore uncouples electron transport and mitochondrial respiration from ATP synthesis. FCCP treatment has been shown to increase O_2_ consumption to the maximum in various cell types^18^, thus enables us to measure the reserve/spare respiratory capacity of the cells. The spare capacity signifies the amount of extra ATP that can be produced by oxidative phosphorylation in case of a sudden increase in energy demand, and it is calculated as the difference between the FCCP-induced OCR and the basal OCR. It is an indirect measure of the cell’s adaptive capacity to metabolic stress^19^. FCCP treatment resulted in an increase of OCR levels in NHFs and the derived KiPS but with a lower extent in iPSCs. Furthermore, we observed that the spare respiratory capacity of differentiated KiPS is higher than that observed for the iPSC lines (Fig. 1B), consistent with the idea that the spare capacity reflects the cell’s propensity to differentiate^18^. We observed that the spare capacity of KiPS-40 was lower than that of KiPS-20, indicating that even though KiPS-40 cells are derived from the iPSCs whose bioenergenic profiles are very similar to that from a younger donor’s iPSCs, their mitochondrial function is in fact suboptimal.

Taken together, these results suggest that iPSCs depend less on OXPHOS than the fibroblasts and KiPS. Interestingly, the iPSCs showed low basal O_2_ consumption rates, and are considerably less sensitive to FCCP. These results suggest that the mitochondria in pluripotent cells, although functional, are less active. Therefore, the iPSCs and their differentiated counterparts are metabolically distinct. Importantly, the metabolic performance of mitochondria iPSCs do not necessarily predict the mitochondrial fitness in its derived KiPS.

### Treatment of KiPS with plant extracts restores their mitochondria function

KiPS-40 derived from the older donor’s iPSCs show suboptimal mitochondrial function (Fig. 1). We therefore wished to determine if treating KiPS-40 with a unique plant extract that is known to potentially protect mitochondria will increase the aerobic metabolism of the KiPS. A mix of plants extracts with 1% Jasminum Officinale Flower & Palmaria Palmata extract and 1% hydrolyzed soy protein extract (“JPPS extract”) was developed. Jasminum Officinale Flower & Palmaria Palmata extract is composed of the extract from Jasminum Officinale flowers (Jasmine) and Palmaria Palmata, a red algae (JPP). Originally derived to target SKPs (skin-derived precursors from the dermis), the treatment of 1% JPP on SKPs increases the embryonic stem cell specific expression markers and leads to better RHE morphology derived from these cells (patented results). Hydrolyzed soy protein is an extract of Innovative *Glycine max* (soybean) rich in peptides biomimetic to respiratory chain proteins. Hydrolyzed soy protein stimulates the Cytochrome c Oxidase in vitro, resulting in a durable energetic increase, necessary for a high metabolism performance and protection without inducing oxidative damage (data not shown). When mixed, the complete JPPS extracts significantly boosted the basal and max OCR and the spare respiratory capacity in KiPS-40, whereas individually, either the Jasmine/red algae extract had very subtle impact on these studied parameters. (Fig S1A). To further assess its efficacy, we compared the effects of adding the JPPS extract–either as a complete mix, or as separate ingredients–to that of ZNL005, a known agonist of a mitochondrial respiration stimulator, the peroxisome proliferator-activated receptor-gamma coactivator (PGC)-1 α. We found that only the complete JPPS extract induced the expression of PGC-1α as potently as ZNL005, if not more (Fig S1B). Therefore, we used the complete JPPS extract in all subsequent experiments.

**Figure 2:**
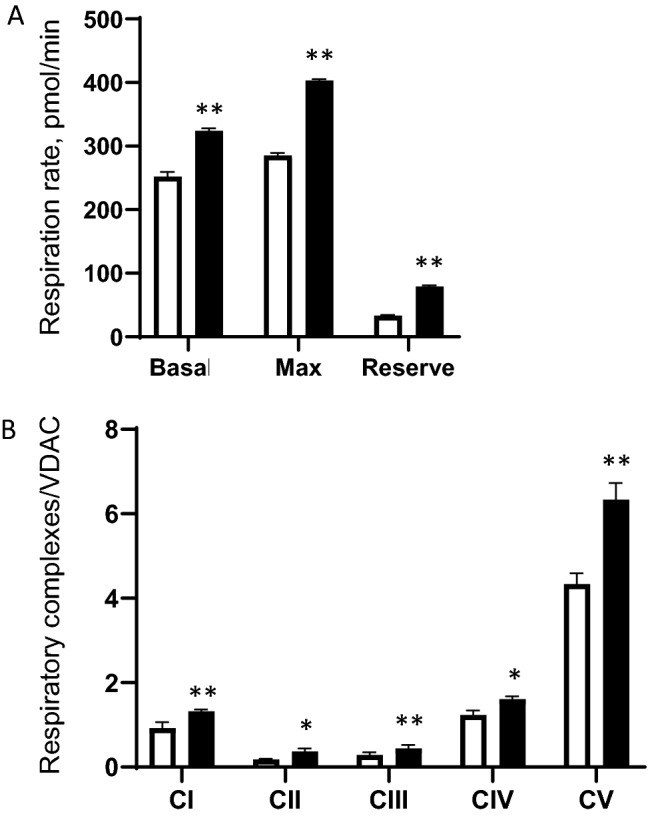
Treatment of KiPS cells with plant extract improved their mitochondrial activity. (**A**) Seahorse analysis of the metabolic features of KiPS-40. Non-treated controls (white bars) or treated KiPS-40 with 1% Jasminum Officinale Flower extract & Palmaria Palmata extract, 1% hydrolyzed soy protein extracts (black bars) at day 4 after the beginning of their differentiation and for 4 days. Basal OCR, maximum respiration in the presence of FCCP and spare respiratory capacity (difference between the FCCP-induced OCR and the basal OCR) was determined. (n = 2 experiments and 5 replicates) (**B**) Quantification of mitochondrial complexes by WES analysis of respiratory complexes. Statistical significance was determined by a Student’s *t* test. **P < 0.01 and *P < 0.05, errors bars: SEM.

Four days after differentiation ensued, KiPS-40 cells were treated for 4 more days with the complete JPPS extract. We observed significant increase in the levels of both basal and maximal respiration rates induced by FCCP treatment (Fig. 2A). To test if the augmented mitochondrial activities in JPPS extract-treated KiPS-40 could be attributed to an increase in respiratory chain complexes expression, we assessed the quantities of mitochondrial complexes I, II, III, IV and V in treated and non-treated KiPS-40 cells using the WES technology, a gel- and blot-free Western blot assay method. Indeed, when compared to non-treated cells, mitochondrial complex I, IV and V protein levels in treated KiP-40 were increased by 44%, 30% and 46% respectively (Fig. 2B). Furthermore, complex II and III show more pronounced protein expression increase, by 105% and 54% respectively (Fig. 2B). These data suggest that treatment with the complete JPPS extract induced mitochondrial respiratory complex biogenesis responsible for the increase in mitochondrial respiratory reserve and thus, improving the mitochondrial fitness of the KiPS-40.

### Capability of iPSC-derived Keratinocytes to synthesize epidermal differentiation markers

**Figure 3:**
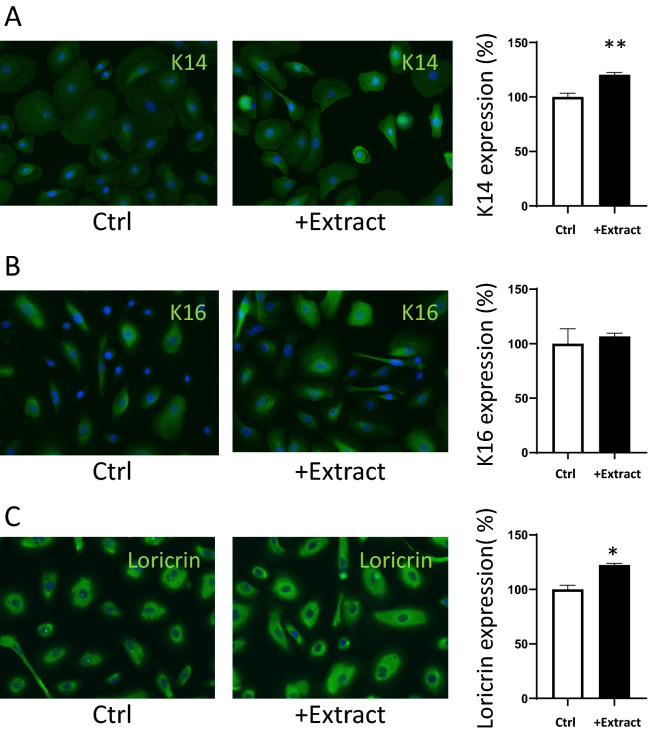
Treatment of KiPS cells with plant extract improved their terminal differentiation. (**A**) Keratin 14 protein immunofluorescent staining in KiPS cells after 48 h of treatment. KiPS-40 at day 8 post differentiation were treated, or not, in duplicate, with 1% JPPS (Extract) directly diluted in the culture medium, twice a day, for 48 h. Quantification of keratin 14 was done using Volocity software. (**B**) Keratin 16 protein immunofluorescent staining in KiPS cells after 48 h. (**C**) Loricrin protein immunofluorescent staining in KiPS cells after 48 h. Statistical analyses are represented as black and white bars (mean ± sem; n = 3; *P < 0.05, with Student’s *t*-test).

Having demonstrated that the mitochondrial function from KiPS-40 can be boosted by our JPPS extract, we wish to test the feasibility of using these treated keratinocytes to generate RHE, and define the minimum quantity of KiPS required to yield RHE of optimal quality. To do so, we examined whether these cells were able to express epidermal differentiation markers and how the addition of JPPS extract impacts their expression. To address this question, we diluted the JPPS extracts directly into the culture medium of the KiPS-40 cells, twice a day for 48 h, and studied the expression of several cellular markers: keratin 14 (Fig. 3A), keratin 16 (Fig. 3B) and loricrin (Fig. 3C) in both treated and non-treated KiPS-40 cells. Keratin-14 expression marks the proliferative capacity in undifferentiated keratinocytes; loricrin is found in terminally differential epidermal cells. We observed significant increases in keratin 14 and loricrin protein, but not Keratin 16, which is typically associated with hyperproliferating keratinocytes in injured or diseased skin. Therefore, the JPPS extract treatment increases the proliferative capacity and enhances the terminal differentiation of KiPS-40, which is adequate to generate RHE.

### Generation of RHE reconstructed using iPSC-derived keratinocytes and normal human keratinocytes cells after treatment with the plant extracts

**Figure 4:**
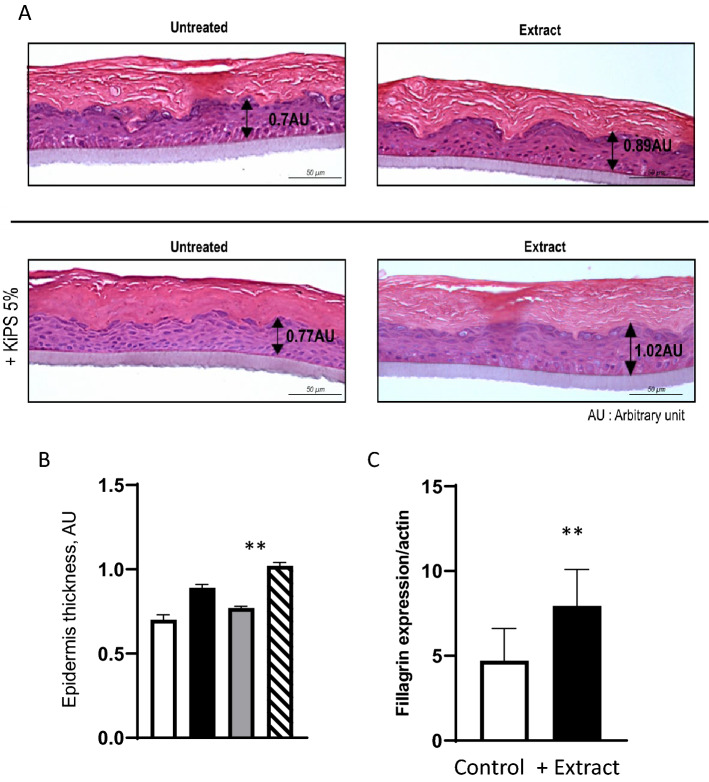
Morphological study of RHE-KiPS after treatment with plant extract. (**A**) Hematoxylin and Eosin staining at Day 13 to study RHE morphology: hematoxylin stains basophilic structures such as the nuclei in blue and purple; Eosin stains acidophilic structures such as the cytoplasm in pink. The upper panel shows RHEs derived from 100% non-treated (left) or pre-treated NHKs (with 1% JPPS extract treatment 48 h before reconstruction). The lower panel shows RHEs derived from non-treated NHKs with the addition of 5% KiPS-40, non-treated (left), or incubated with the 1% JPPS extract added 48 h before the reconstruction commenced (right). (**B**) Quantification of epidermis thickness of non-treated (white bar) or pre-treated with JPPS extract NHK (black bar), non-treated NHKs with the addition of 5% KiPS-40 (grey bar) or treated (dashed bar), To measure the epidermal thickness of, measurements of viable cellular layers were conducted on three reconstructed tissue after H&E staining (Three pictures per condition were taken and 6 measurements of the thickness were calculated per picture. The measurement is the mean of the three pictures. *AU* arbitrary unit. (mean ± sem; n = 3; *P < 0.05, with Student’s *t*-test). (**C**) Quantification of fillagrin by WES analysis. 5% KiPS-40 treated (black bars), and non-treated extract (white bars) were added the day of the reconstruction and RHEs were grown for 13 days, then RHE protein extraction was performed and filaggrin was quantified bars (mean ± sem; n = 3; *P < 0.05, with Student’s *t*-test).

As a final step toward the future use of iPSC-derived keratinocytes whose mitochondrial performance is boosted by JPPS-extract, we generated KiPS-40 based RHE. As a rule of thumb, after 10 To 14 days of growth, a well differentiated RHE should closely resemble the human epidermis marked by a stratified basal layer, spinous layers, granular layers and a stratum corneum. We first reconstructed the RHEs with 100% KiPS-40, but found certain specific epidermal layers were missing and not well-defined (Figure S3). We then tested the combination with 80% NHK derived from a biopsy tissue of a heathy two-year old boy mixed it with 20% KiPS-40. The quality and morphological aspects of the reconstruct epidermis were not improved (data not shown). Eventually, we found that in an RHE including only 5% KiPS with 95% NHK was sufficient to generate an end-product whose tissue morphology was identical to the sample derived from 100% KiPS-40, but the cellular junctions were more numerous, the stratum corneum was more prominent and contained multiple layers (Fig. 4). Encouraged, we decided to use the composition of 95%NHK/5%KiPS-40 for further studies.

To generate RHEs, we seeded the NHKs on inert polycarbonate membrane and cultivated them with or without 5% KiPS-40, in the presence or absence of the complete JPPS extract. As a control, we also included a condition where the NHKs were directly treated or not with the JPPS extract without KiPS-40 addition (Fig. 4).

First, the morphological components were present in all RHEs, but there were notable differences. The RHEs derived from 100% NHKs treated with the complete JPPS extract displayed a 27% increase in tissular thickness compared to the RHE derived from the untreated NHK (Fig. 4A). The addition of 5% KiPS-40 in the composition of RHE promoted even better basal activity, and an increase of 10% in tissue thickness was observed compared to the RHE derived from NHK alone. Treatment of RHE-KiPS with the JPPS extract, however, produced the most striking results. Under treatment, epidermal thickness was increased by 33% compared to the untreated RHE-KiPS, and by 45% compared the non-treated, 100% NHK-derived RHE (Fig. 4B). Thicker stratum corneum reflects greater skin turn-over and therefore a better renewal. No vimentin expression was detected in the RHE-KiPS, suggesting that the initial fibroblast identity was not retained (data not shown).

Interestingly, we observed that in the non-treated RHE-KiPS-40, differentiation markers such as loricrin and filaggrin were reduced by 56% and 53% respectively, indicating that the inclusion of 5% untreated KiPS-40 is insufficient to support the terminal differentiation of the bio-engineered RHE tissue. The incubation with the JPPS extract could partially recue the reduced expression of loricrin and filaggrin of 5% KIPS-RHE when compared to NHK-RHE (Fig. S4). At this stage, we observed no modification of K10 expression (early differentiation marker) in all conditions (data not shown).

Lastly, to ascertain if the impairing mitochondrial respiration has an immediate impact on the RHE differentiation, we treated the RHE with rotenone, a potent inhibitor of NADH reductase in complex I of mitochondrial respiratory chain. Compared the control, rotenone treatment led to 77% or 68% decrease in ATP production in the RHE with KiPS or NHK, respectively (Figure S2A). We also observed a concomitant increase in Filaggrin expression (Figure S2B). This observation implies that rotenone inhibition of the mitochondria compromises the cellular capacity to proliferate when the available ATP reserve is depleted, and consequently drives the cells into a further differentiated state. Taken together, our data from Figs. 3 and 4 indicate that our JPPS extract is important for KiPS differentiation.

### Metabolic characterization of RHE reconstructed with KiPS after treatment with the plant extracts

**Figure 5:**
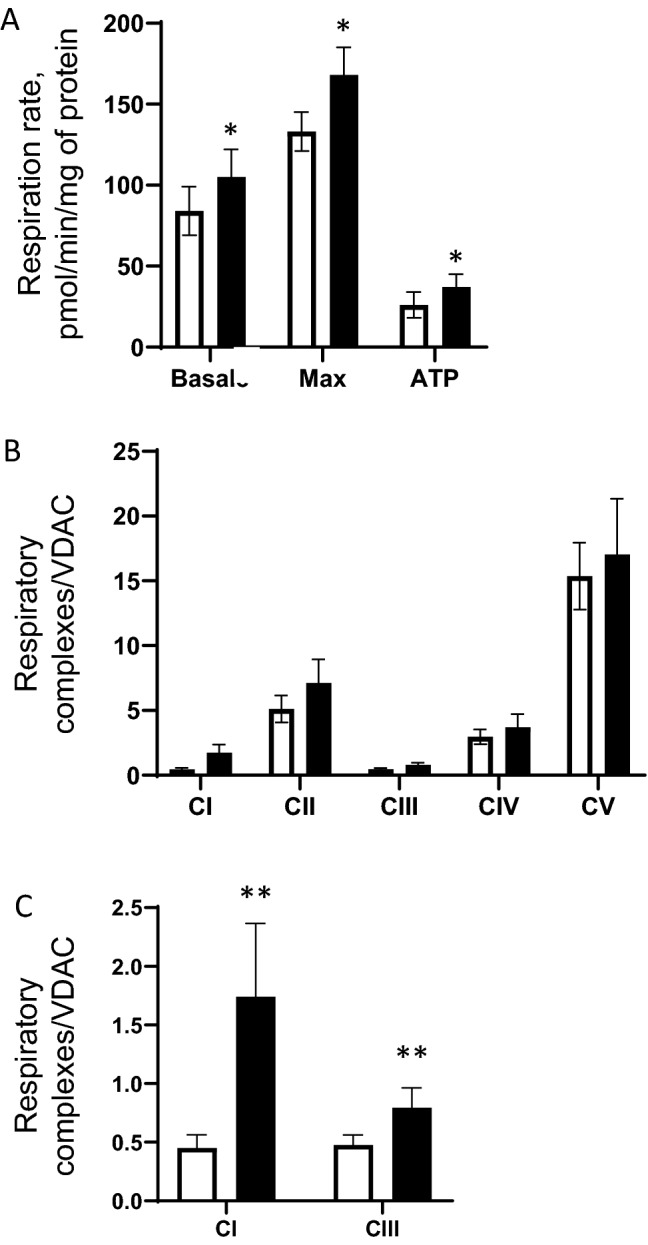
Aerobic metabolism of RHE-KiPS after treatment with plant extract. (**A**) Seahorse analysis of metabolic features of RHE-KiPS at day 13. RHE-control (white bars) or treated with 1% Jasminum Officinale Flower extract & Palmaria Palmata extract, 1% hydrolyzed soy protein extracts (black bars). Statistical significance was determined by Student’s *t*-test, (mean ± sem; n = 3) **P < 0.01 and *P < 0.05, errors bars: SEM. (**B**) Quantification of mitochondrial complexes by WES analysis of respiratory complexes. (**C**) Quantification of mitochondrial complexes I and III. Statistical significance was determined by a Student’s *t* test. **P < 0.01, errors bars: SEM.

In order to investigate whether the RHE reconstructed with 5% KiPS-40 presented adequate mitochondrial activities, we determined the O_2_ consumption rates (OCR). Both basal and max respiratory reserve of the tissue were increased in the treated tissue (Fig. 5A). We further tested if the increase in respiration was due to increased expression of mitochondrial complexes. We found that when treated with the complete JPPS extract, the protein expression of complex I and III were increased by 286% and 67% respectively compared to the non-treated RHE-KiPS (Fig. 5B,C).

In summary, the addition of 5% KiPS with the concomitant treatment of mitochondrial-boosting JPPS complex generated a RHE with an increased epidermal thickness. This may be attributed to the positive modulation on the expression of respiratory complex chain proteins, notably complex I and III, and inhibition of the mitochondrial complex I has a direct impact on the differentiation state of the reconstructed RHE.

## Discussion

We were able to reprogram the human fibroblasts of a 20- and a 40-year old donors into iPSCs, and differentiate them into KiPS to reconstruct RHE tissue. We then analyzed the specific mitochondrial respiratory functions of these derived cell lines and the reconstructed RHEs from these isolated samples. Significant differences in mitochondrial respiratory capacity were found between the young and the old fibroblasts and their iPSC-derived keratinocytes, which underscores the importance to evaluate mitochondrial health and function before embarking on the task to reconstruct 3D skin culture (Fig. 1A). Consistent with previous findings, the mitochondria from the iPSCs derived from either the young or the older donors’ fibroblasts show less basal OCRs and spare respiratory capacity compared to their parental NHFs and derived KiPS, confirming that the reprogramming into iPSCs transform the mitochondria to an immature metabolic state that preferentially relies on glycolysis for ATP generation and thus is more resistant to the perturbations in oxidative phosphorylation. However, despite the similarities in mitochondrial activity profiles in iPSC progenitors, the derived KiPS lines show marked difference. Specifically, mitochondria from KiPS-40 have lower oxygen consumption rates and less spare respiratory capacity (Fig. 1B). Since the biopsy sample is derived from a unique patient, it is difficult to attribute such decreased mitochondrial activities to any discrete cause, such as natural aging or genetic predisposition, yet this observation prompted us to test our signature natural extract (JPPS) enriched in compounds known to protect mitochondria, which yielded encouraging results as such treatment improved various mitochondrial performance parameters and renders the derived KiPS fully competent for RHE reconstruction when using maximal 5% KiPS(Fig. 4).

The mitochondrial function was repressed to the same extent in the iPSCs derived from both the old and the young donor. This observation thus concurs with a recent study reporting that iPSCs contain immature and inactive mitochondria^9^. It has been proposed that dedifferentiation of epidermal keratinocytes from elderly patients was driven by the induction of stemness transcription factors and associated with suppression of senescence/apoptosis pathways, suggesting that reprograming may be a tool to rejuvenate old cells^12,20^. However, since we observed that KiPS derived from the 40-year old donor’s iPSCs displayed reduced mitochondria respiratory capacity compared to the young ones, we wish to propose that the iPSCs and their differentiated counterparts are metabolically different, and that these metabolic parameters also represent important features for stem cell identity. Furthermore, a previous study showed that when differentiated back into fibroblasts, iPSCs from old donor cells have rejuvenated extended life spans and characteristics of young proliferative embryonic fibroblasts^12^. Our results impose certain caution to this conclusion. We emphasize that it is of therapeutic interest to examine the mitochondrial bioenergetic profiles pre- and post-differentiation process, so to reveal a potentially occult aspect of the cell’s aging phenotype. Consistent with our proposal, spare respiratory capacity seems to be an important parameter to define cell’s propensity to differentiate^19^.

To re-energize the KiPS derived from the old donor for tissue reconstruction, we sought for plant extracts capable of optimizing mitochondrial function. Extracts from plants and natural compounds have been historically used in traditional medicine as ointments or for ingestion, but the effects of most of these compounds have yet to be determined. From the patent-protected compounds under development in our laboratory, we chose to mix the plant extract from Jasminum officinale flowers and Palmaria Palmata with hydrolyzed soy proteins extract. It is capable of stimulating mitochondrial respiration as potently as the known PCG-1α agonist ZNL005. We found this unique formula not only increased mitochondria respiration activity, but also respiratory complexes biogenesis in KiPS derived from the older donor’s issue, suggesting that they potentially contain active compounds to remodel the mitochondrial proteome. Agents that protect cells against loss in mitochondrial spare respiratory capacity, or that induce mitochondrial respiratory complexes biogenesis, are thought to have therapeutic potential for treating numerous pathologies^21^. Our findings here suggest that natural compounds targeting mitochondrial respiratory reserve could also hold promise as an anti-aging strategy. Indeed, including only 5% of such treated KiPS in the tissue reconstruction process is sufficient to elicit a beneficial effect, manifested as thicker and thus healthier epidermal layer in the RHEs.. In conclusion, by improving the spare respiratory capacity in the KiPS, and including only minimum amount of these cells in the RHE generation process, we were able to obtain a KiPS-RHE that are well differentiated, fully functional and presenting good respiratory reserve. These RHE are a perfect tool for studying aerobic metabolism and screening for mitochondrial activator ingredients. Taken together, our protocol with the plant extracts treatment has many advantages to derive proper keratinocytes from iPSCs for future 3D skin reconstruction.

## Methods

### Chemicals and antibodies

All chemicals were purchased from Sigma-Aldrich (Saint Quentin Falavier, France). The antibodies used in the study were Anti-keratin 14 (Abcam, Cambridge, MA, USA, mouse monoclonal), Anti-keratin 16 (Abcam, Cambridge, MA, USA, mouse monoclonal), Anti-loricrin (Abcam, Cambridge, MA, USA) mouse monoclonal, Alexa Fluor 488 donkey anti-mouse (Thermofisher scientific, Montigny le Bretonneux, France).

### Plan extract preparation and characterization

All the plant extracts were produced by Ashland (Ashland, Sophie Antipolis, France).

### Skin sample and cell culture

The primary skin cell cultures were established individually from an abdominal skin biopsy obtained from a 20 and 40-year-old Caucasian healthy female undergoing plastic surgery, with the informed consent of the donors whose identities were kept strictly anonymous. Experimental design was conducted with respect to the ethical permissions. The principal requirements of the Declaration of Helsinki were used as the guidelines to protect the rights, safety and the well-being of the subjects participating in the study. Before initiating the studies, the investigator had obtained written consent from the participants and full approval from the Freiburg Ethics Commission International for the protocol(s), protocol amendment(s), if applicable. All participants whose skin biopsies were used for this research provided their written informed consent to participate in this study, and for the data to be used for research purposes. After removal of the hypodermis, the skin biopsy was rinsed twice with PBS, followed by ethanol/ PBS (2/3:1/3) and a final wash in PBS. Ashland agreement DC 2011-1323.

### Normal human fibroblast (NHF) culture

NHF were extracted from normal human dermis. NHK were cultivated in serum-free DMEM medium containing 20% FBS, 2 mM glutamine, 100 U/ml penicillin, and 100 µg/ml streptomycin at 37 °C in a humidified atmosphere containing 5% of CO_2_ as previously described^4^.

### Derivation of iPSCs

Fibroblasts from the 20- or 40 year old donor were transduced using the CytoTune–iPS Reprogramming Kit (Thermofisher Scientific, Montigny le Bretonneux, France) based on the Sendai lentiviral system. Modified and non-transmissible form of the virus was produced to deliver and express four transgene-encoding: OCT3/4, SOX2, KLF4, and c-MYC. Following previously published procedures^12^, pluripotency of the generated iPSC lines was confirmed by both in vitro embryoid bodies (EB)-based differentiation and teratoma formation.

### IPS cell culture

All pluripotent stem cells were cultivated on mitotically-inactivated mouse embryonic fibroblasts (MEFs) grown on Matrigel (BD Bioscience, San Jose, CA, USA)-coated plates, using KO-DMEM medium (Thermofisher scientific, Montigny le Bretonneux, France) containing 20% knock-out serum replacement (KSR, Thermofisher scientific, Montigny le Bretonneux, France), 8 ng/ml bFGF (PeproTech, Neuilly sur Seine, France).

### IPSC differentiation into keratinocytes

To differentiate iPSCs into keratinocytes, small clumps of iPSCs were sub-cultured on Matrigel (BD Bioscience, San Jose, CA, USA) in MEF-conditioned media for 24 h.

iPSCs were then incubated in defined keratinocyte serum-free medium (DKSFM; Thermofisher scientific, Montigny le Bretonneux, France) supplemented with 1 μM all-trans retinoic acid Sigma Aldrich, Saint Quentin Falavier, France) and 10 ng/mL bone morphogenetic protein 4 (BMP4; R&D Systems, Mineapolis, USA) for 7 days. The cells were subsequently cutltured in EpiLife medium (Thermofisher scientific, Montigny le Bretonneux, France) for 7 more days before the final harvest and analyses. iPSC-derived keratinocytes (KiPS) were cultured in Serum Free Medium Epilife, supplemented with S7 (Thermofisher scientific, Montigny le Bretonneux, France) for 7 days before analysis.

### KiPS culture

KiPS were grown in Serum Free Medium Epilife, supplemented with S7 (Thermofisher scientific, Montigny le Bretonneux, France) and 35 μg/ml of Gentamycin (Euromedex, Souffelweyersheim, France). Cells were maintained at 37 °C in a humidified atmosphere containing 5% of CO_2_.

### KiPS treatment

Sham control and treated KiPS samples were prepared in duplicate, with 1% Jasminum Officinale Flower extract & Palmaria Palmata extract + 1% Hydrolyzed soy protein extracts directly diluted in the culture medium, twice a day.

### Seahorse analysis of IPS cells, KiPS and normal human fibroblasts

Mitochondrial respiration was assessed using a Seahorse XF96 extracellular flux analyzer (Agilent, les Ulis, France). The iPSCs were dissociated into single cells and 4 × 10^4^ cells were seeded onto an XF96 cell-culture microplate coated with Matrigel. The next day, the medium was replaced with XF assay medium, followed by sequential injection of 1 μM oligomycin A, 1 μM FCCP, 2 μM antimycin/rotenone (Sigma -Aldrich, Saint Quentin Falavier, France). The results were normalized to the total protein content in each well, which was determined using DC Protein Assay Reagent (Sigma -Aldrich, Saint Quentin Falavier, France). For the other cell types, the cells were seeded onto an XF96 cell-culture microplate 4 × 10^4^ /well and the assay was done using the same protocol.

### Immunofluorescence assays

After treatments, KiPS were rinsed with PBS and fixed with 3.7% paraformaldehyde for 10 min at room temperature. Cellular membranes were incubated for ten minutes in PBS with 0.1% Triton X-100 (Thermofisher scientific, Montigny le Bretonneux, France) solution for permeablization. Unspecific binding sites were blocked with 1% BSA (Sigma-Aldrich, Saint Quentin Falavier, France) solution for 30 min. Cells were then incubated with the primary antibody, under agitation, at 4 °C. After three PBS-washes, the fluorescent secondary antibody was applied, in the dark, under agitation, at room temperature. After three other washes with PBS, the cell nuclei were stained with 4',6'-diamidino-2-phenylindole (DAPI, Thermofisher scientific, Montigny le Bretonneux, France) at 0.3 µM for 5 min and slides were mounted in Fluoromount-G (Electron Microscopy Sciences, Hatfield, PA, USA). Detection was managed and examined using a Zeiss Axiovert 200 M microscope with a 20 × objective. Photos were taken with a Q imaging EXI blue camera coupled to Volocity acquisition software (Improvision).

### Reconstruction of the epidermis equivalent model (RHE)

Epidermal model is a reconstructed human epidermis equivalent developed by the Advanced Skin Research and Bioengineering Department at Ashland Global Skin Research Center, Sophia Antipolis, France (Capallere et al. 2018). To obtain the RHE, normal human primary keratinocytes (NHK) were isolated from skin explants from a healthy 2-year old donor after surgery. Briefly, the biopsy sample was incubated overnight at 4 °C in Dispase II (Sigma -Aldrich, Saint Quentin Falavier, France) to remove epidermis from dermis, the remaining cells were then dissociated by Trypsin (Sigma -Aldrich, Saint Quentin Falavier, France), centrifugated and resuspended in culture medium (MCDB 153 modified (Sigma-Aldrich, Saint Quentin Falavier, France). To reconstruct RHEs, the isolated NHKs and cultured KiPS-40 were seeded on a 0.5 cm^2^ inert polycarbonate membrane (Nunc, Denmark) in a chemically defined medium and were air-lifted for several days at 37 °C under 5% CO_2_.

### Morphological observation following H&E stain and thickness measurement

For RHE preservation, the tissue were fixed for 4 h in buffered 10% formalin. Samples were serially transferred to ethanol baths with progressively increasing concentration to remove water, then followed by two baths of xylene to remove the alcohol, and finally embedded in molten paraffin wax. Embedded RHEs were then sectioned with a microtome (Thermofisher scientific, Montigny le Bretonneux, France) into 4 μm thick and placed on slides. Sections were deparaffinized and rehydrated with several successive xylene, alcohol and water baths. Slides were then stained for 3 min in a 50% Hematoxylin bath, rinsed for 5 min in water and then stained again for 2 min in a 60% Eosin bath. After several successive alcohol and xylene baths, slides were mounted in Eukitt (Dutscher, Brumath, France) and examined using an Eclipse E600 microscope (Nikon) with a 20 × objective. Photos were taken with a Qimaging EXI blue camera and processed by using the Q-Capture Pro 7 (QImaging, Tucson, USA) acquisition software.

To measure the epidermal thickness, three measurements of viable cellular layers were conducted on three reconstructed tissue after H&E staining. Three pictures were taken and 6 measurements of the thickness were taken per picture.

### Automated capillary electrophoresis western analysis

KiPS or RHE were lysed in whole cell extraction buffer (25 mM Tris HCL buffer pH 7.5; 0.25 M saccharose; 0.2 mM MgSO_4_; 20 mM EDTA; 0.4% Triton X-100; 2 mM DTT; 5 µg/ml leupeptin; 0,4 mM PMSF). Cell lysates were sonicated for 5 cycles of 8 s ON/30 s OFF with a Bioruptor Pico Ultrasonicator (Diagenode, France) at 4 °C, then spun down for supernanant harvest. Protein concentration was determined by the DC protein assay (Bio-Rad laboratories, Hercules, CA).

Mitochondrial complexes protein levels were determined by capillary electrophoresis immunoassay by following the Wes user guide from Protein Simple (Protein Simple, San Jose, CA, USA) and primary antibody dilution were determined. Anti OXPHOS mouse (45-8199, Thermofischer) 1/50, anti-VDAC1 rabbit (NB100695, Novus Biologicals Europe) 1/200, anti-NDUFB8 (C I) rabbit (NBP1-88859; Novus Biologicals Europe) 1/100, Anti-UQCRC2 (C III) rabbit (NBP1-80861; Novus Biologicals Europe) 1/50, anti PGC1 rabbit (NBP1-04676, Novus Biologicals) 1/80, anti -tubulin rabbit (NB600-936 ;Novus Biologicals) 1/1000.

After plate loading, the electrophoresis and immunodetection steps took place in the capillary system (Protein Simple Wes, Protein Simple, San Jose, CA, USA) and were fully automated with instrument default settings. Peak areas were determined using Compass software (Protein Simple) and normalized to VDAC (loading control) (NB100695, Novus Biologicals Europe).

### Seahorse analysis of RHE

XF24-ExtracellularFluxAnalyzer analyzer (Agilent, les Ullis, France) was used to measure the change of oxygen consumption rate (OCR). XF Islet Capture Microplates (Agilent, les Ullis, France) were used for measurements. These plates contain a net that prevent the oxygen sensor to touch the RHE. The basal part of the epidermis was put on the net. This orientation was essential for the measurement to assure that the part of the RHE which respires was close to the oxygen sensor. The RHE were covered with XF assay medium, followed by sequential injection of 4 μM oligomycin A, 4 μM FCCP, 2 μM antimycin/rotenone (Sigma -Aldrich, Saint Quentin Falavier, France). The results were normalized to the total protein content in each RHE, using lysis buffer (25 mM Tris HCL buffer pH 7.5; 0.25 M saccharose; 0.2 mM MgSO_4_; 20 mM EDTA; 0.4% Triton X-100; 2 mM DTT; 5 µg/ml leupeptin; 0,4 mM PMSF) and DC Protein Assay Reagent.

### Statistical analysis

Results were expressed as mean ± SEM and analyzed using GraphPad Prism 8.4.3. Software. The student tests were used to compare data sets.

## Supplementary Information

Supplementary Information.

## Data Availability

The data that support the findings of this study are available from Dr Capallere but restrictions apply to the availability of these data, which were used under license for the current study, and so are not publicly available. Data are however available from the authors upon reasonable request and with permission of Dr Capallere.
