# Supplementary material for: Reconstruction of functional human epidermis equivalent containing 5%IPS-derived keratinocytes treated with mitochondrial stimulating plant extracts
Source: Sci Rep. 2022 May 31;12:9073. doi: 10.1038/s41598-022-13191-4 (PMC9156774; doi:10.1038/s41598-022-13191-4)
Supplement: Supplementary file 1 — Supplementary Information. [file 41598_2022_13191_MOESM1_ESM.pdf]

Reconstruction of functional human epidermis equivalent containing 5 %IPS-derived keratinocytes treated with mitochondrial stimulating plant extracts.

Marielle Moreau[1§], Christophe Capallere[2§], Laurent Chavatte[3], Christelle Plaza[2], Céline Meyrignac[2], Karl Pays[1], Bruno Bavouzet[1], Jean-Marie Botto[2], Carine Nizard[1], Anne-Laure Bulteau*[1] orcid.org/0000-0002-3315-5100

1. LVMH Recherche. Life Science Department, 185 Avenue de Verdun, 45800. Saint Jean de Braye. France.

2. Ashland, Global Skin Research Center, Advanced Skin Research & Bioengineering Dept, Sophia Antipolis, France.

3. CIRI, Centre International de Recherche en Infectiologie, CIRI, 69007 Lyon, France. Institut National de la Santé et de la Recherche Médicale (INSERM) Unité U1111, 69007 Lyon, France. Ecole Normale Supérieure de Lyon, 69007 Lyon, France. Université Claude Bernard Lyon 1 (UCBL1), 69622 Lyon, France. Unité Mixte de Recherche 5308 (UMR5308), Centre national de la recherche scientifique (CNRS), 69007 Lyon, France.

*Address correspondence to: Anne-Laure Bulteau. LVMH Recherche. Life Science Department, 185 Avenue de Verdun, 45800. Saint Jean de Braye. France.

Email: abulteau@research.lvmh-pc.com Phone: 33-02-38-25-84-19.

[§] These two authors contribute equally

Supplementary Methods

ATP determination

Total ATP was quantified using a luminescence ATP detection assay kit (Abcam). Assay was performed using supplier recommendations.

Rotenone treatment of RHE.

NHF and KiPS were cultivated in serum-free medium with a proprietary formula and maintained at 37°C in a humidified atmosphere containing 5% of CO_2. The cells were then harvested and seeded on an inert polycarbonate membrane (0.5 cm^2 insert, Nunc) and were air-lifted for 12 days on a chemically defined medium at 37°C in a humidified atmosphere containing 5% of CO_2.

After 12 days of culture, RHE were treated with rotenone for 3h. After removing culture medium containing rotenone, a fresh culture medium was added for 48 hours, once a day.

Immuno-staining of RHE

Sections were deparaffinized and rehydrated with several successive xylene, alcohol and water baths. Then, an unmasking protocol was performed first by microwave exposure at 600 W in a citrate buffer of 0.01 M pH 6 (Sigma) until boiling, followed by 0.25% pepsin (Zymed, Invitrogen) digestion for 15 minutes at 37°C. After a PBS wash and saturation of unspecific sites with a solution of 5% BSA (Sigma) for 30 minutes, the primary antibody was applied and the slides were incubated under agitation, at room temperature, in a humid room. After rinsing the slides with PBS, the sections were mounted in Fluoromount-G* (Electron Microscopy Sciences). Detection was managed and examined using a Zeiss Axiovert 200M microscope with a 20x objective. Photos were taken with a Qimaging* EXI blue camera coupled to

Volocity* acquisition software (Improvision). The antibodies used were - Anti-loricrin (Abcam) rabbit polyclonaland -and Anti-filaggrin (Santacruz) mouse monoclonal.

Three photos per condition were analyzed with Volocity* image analysis software (Improvision), that permits the selection of the representative zone based on fluorescence intensity. The results obtained were the sum of green or red pixel intensities in the selected zone. Finally, for each photo, the sum obtained was adjusted by considering the area of the examined epidermis zone.

Figure S1. Treatment of KiPS cells with plant extract improves their mitochondrial activity

A) Seahorse analyses of metabolic features of KiPS derived from a 40-year old donor.

KiPS from the 40-year old donor (KiPS-40), control (white bars) or treated with the complete JPPS extracts (black bars), 1% Jasminum Officinale Flower extract & Palmaria Palmata extract (dark grey bars) or 1% hydrolyzed soy protein extracts (grey bars) at day 4 after the beginning of their differentiation and for 4 days. Basal OCR, Max, maximum respiration in the presence of FCCP and Reserve, spare respiratory capacity B) Quantification of mitochondrial transcription factor PGC1-α by WES analysis. KiPS from the 40-year old donor (KiPS-40), control (white bar) or treated with the complete JPPS extract (black bar), 1% Jasminum Officinale Flower extract & Palmaria Palmata extract (dark grey bar), 1% hydrolyzed soy protein extracts (grey bar) or PGC1-α activator ZLN005 at 2.5 μM (dashed bar) at day 4 after the beginning of their differentiation and for 4 days. Statistical significance was determined by a Student's *t* test. $**P < 0.01$ and $*P < 0.05$, errors bars: SEM.

Figure S2. Rotenone treatment of RHE

After 12 days of culture, RHE were stressed, or not, with systemic application of rotenone for 3h. After removing culture medium containing rotenone, a new culture medium was added for 48 hours. A) ATP measurement. Non-treated controls or KiPS-40 (white bars), treated with rotenone (black bars), treated with 1% Jasminum Officinale Flower extract & Palmaria Palmata extract, 1% hydrolyzed soy protein extracts (grey bars) or treated with 1% Jasminum Officinale Flower extract & Palmaria Palmata extract, 1% hydrolyzed soy protein extracts and rotenone (dashed bars). and B) Filaggrin immunostaining. Statistical significance was determined by a Student's *t* test. $^{**}P < 0.01$ and $^{*}P < 0.05$, errors bars: SEM.

Figure S.3. Morphological study of RHE-KiPS made with different number of KiPS

Hematoxylin and Eosin staining for the observation of RHE morphology: Hematoxylin stains in blue/purple basophilic structures such as the nuclei and Eosin stains in pink acidophilic structures such as the cytoplasm. 100 % KiPS from the 40-year old donor (KiPS-40) were used to reconstruct RHE. A) RHE morphology at day 7. B) RHE morphology at day 10.

Figure S4. Improved terminal differentiation of RHE-KiPS after treatment with plant extract

Immunostaining and Hematoxylin /Eosin staining of RHE reconstructed with 100 % NHK (White bars), or with 5 % KiPS non-treated (black bars) or treated with the complete JPPS extract (grey bars). A. Filaggrin staining and Loricrin staining. B. Immunostaining quantification. RHE were analyzed at day 13 after reconstruction. Statistical significance was determined by a Student's *t* test. $^{**}P < 0.01$.

Figure S1

A

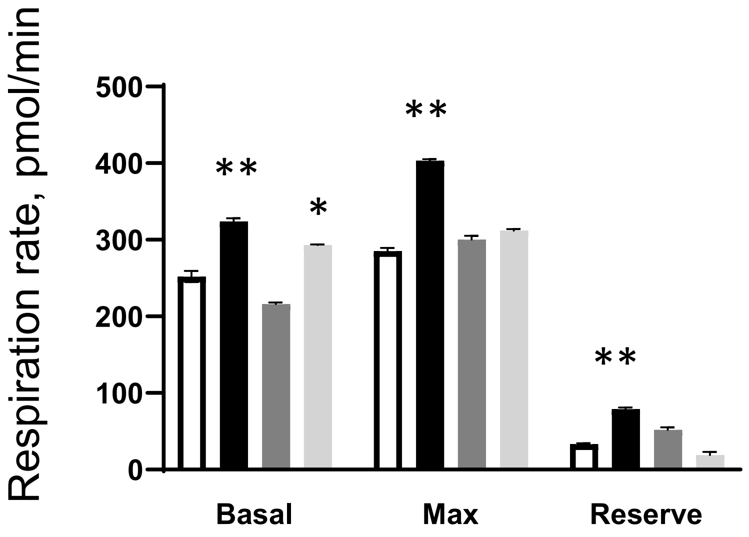
Respiration rate, pmol/min
500
400
300
200
100
0
**
*
**
**
Basal
Max
Reserve

B

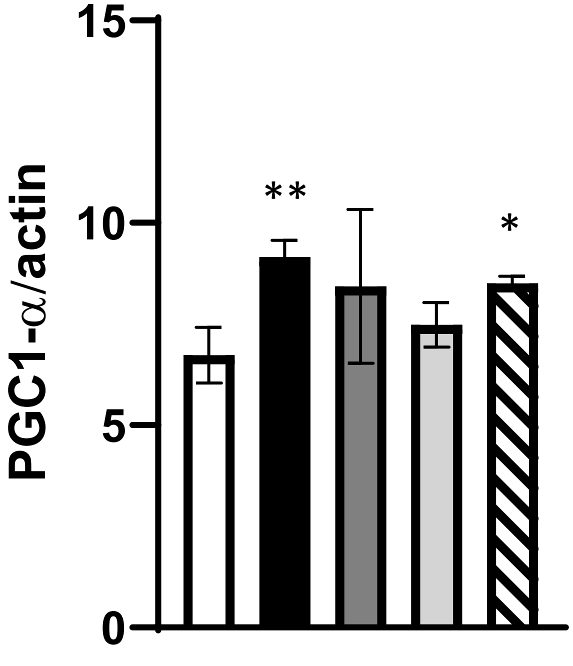
PGC1-α/actin
15
10
5
0
**
*

Figure S2

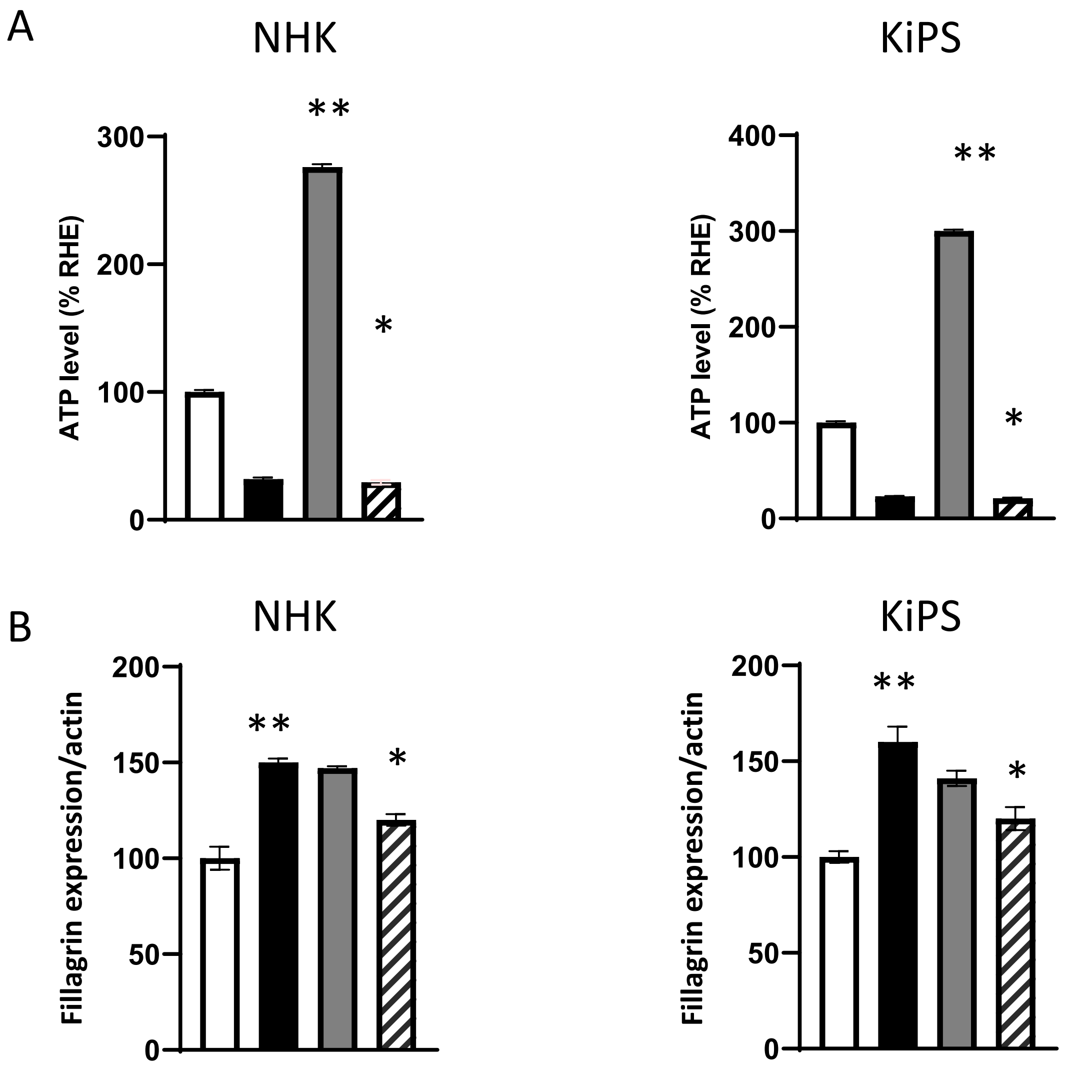
A
NHK
KiPS
ATP level (% RHE)
300
200
100
0
400
**
*
B
NHK
KiPS
Fillagrin expression/actin
200
150
100
50
0
**
*

Figure S3

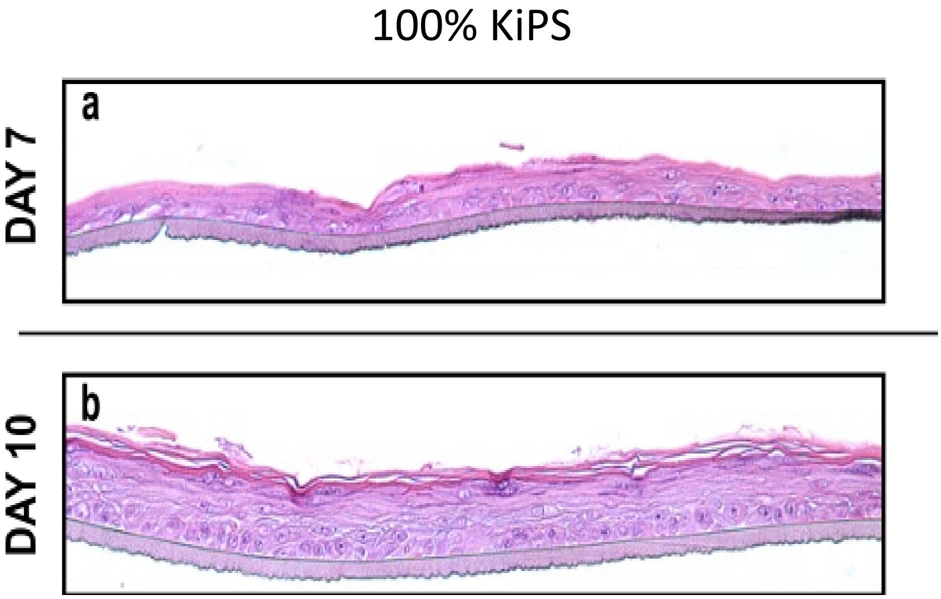

Figure S4

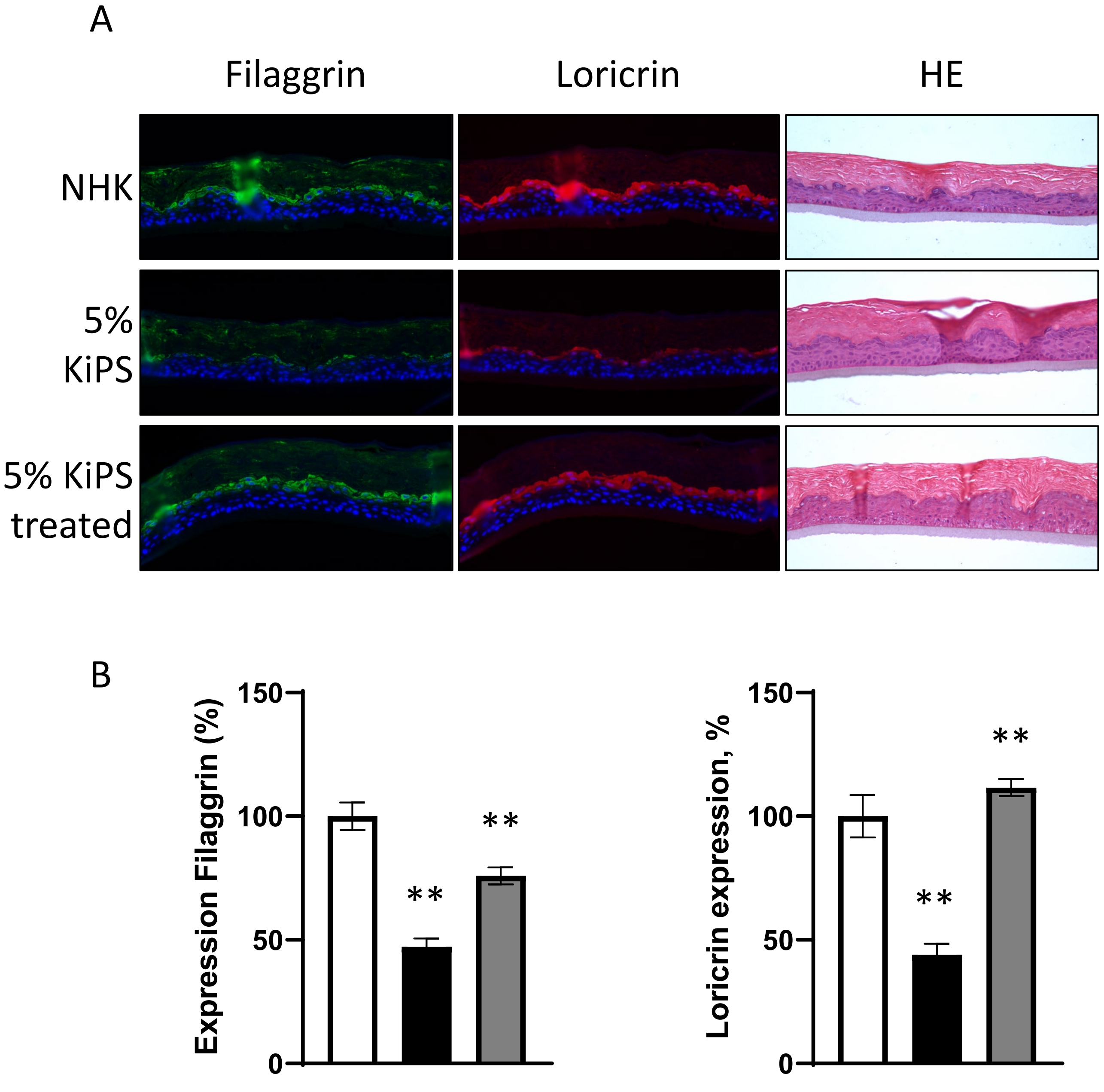
A
Filaggrin
Loricrin
HE
NHK
5%
KiPS
5% KiPS
treated
B
Expression Filaggrin (%)
150
100
50
0
**
**
Loricrin expression, %
150
100
50
0
**
**
